# Coordinate transformation approach to social interactions

**DOI:** 10.3389/fnins.2013.00147

**Published:** 2013-08-21

**Authors:** Steve W. C. Chang

**Affiliations:** ^1^Center for Cognitive Neuroscience, Duke Institute for Brain Sciences, Duke UniversityDurham, NC, USA; ^2^Department of Psychology, Yale UniversityNew Haven, CT, USA

**Keywords:** social interactions, coordinate transformation, reference frames, social decision making, reward, agency, theory of mind (ToM), reinforcement (psychology)

## Abstract

A coordinate transformation framework for understanding how neurons compute sensorimotor behaviors has generated significant advances toward our understanding of basic brain function. This influential scaffold focuses on neuronal encoding of spatial information represented in different coordinate systems (e.g., eye-centered, hand-centered) and how multiple brain regions partake in transforming these signals in order to ultimately generate a motor output. A powerful analogy can be drawn from the coordinate transformation framework to better elucidate how the nervous system computes cognitive variables for social behavior. Of particular relevance is how the brain represents information with respect to oneself and other individuals, such as in reward outcome assignment during social exchanges, in order to influence social decisions. In this article, I outline how the coordinate transformation framework can help guide our understanding of neural computations resulting in social interactions. Implications for numerous psychiatric disorders with impaired representations of self and others are also discussed.

## Introduction

The brains of many animals have evolved to deal with an increasing demand for complex social interactions. Interacting with other members in large social groups requires neural representations to be dynamically updated with respect to oneself as well as with respect to other individuals in order to adjust ongoing social behaviors. Even a simple interaction with another individual requires an accurate tracking of actions and outcomes referenced to self and others. Explorations into how the brain computes information necessary to guide social behaviors can thus reveal ecologically valid insights into neural mechanisms underlying complex cognition that might not be tractable otherwise. One might even argue that probing the brain function using socially relevant behavioral tasks is a preferred way to unlock the mystery of “high-level” cognition in highly social species. Furthermore, a failure to accurately represent self and others can result in atypical social behaviors like those that are striking in autism (Baron-Cohen, 1988) and Williams syndrome (Jones et al., 2000), as well as in schizophrenia (Jeannerod, 2008), borderline personality disorders (Bender and Skodol, 2007) and psychopathy (Hare, 1999). Investigating the neural mechanisms underlying social interactions will therefore provide critical clues toward characterizing the neural basis of a surprisingly large number of neuropsychiatric disorders that are accompanied by social deficits.

Since the early beginning, a major focus in the field of systems neuroscience has been to understand how perception and action are encoded by individual neurons (Goodale and Milner, 1992), and how these signals are transformed across different neural networks (Salinas and Abbott, 1995; Colby, 1998; Colby and Goldberg, 1999). A *coding scheme* of a neuron conveys precise computational principles used in transforming a signal encoded under one coordinate system into a signal encoded under a different coordinate system (Andersen et al., 1993; Pouget and Sejnowski, 1997; Pouget and Snyder, 2000; Snyder, 2000; Groh, 2001; Crawford, 2004). An immense body of work has enhanced our understanding of sensorimotor behavior, such as motor planning and attention, by framing these computational tasks in terms of coordinate transformations.

Here I propose that applying a coordinate transformation model to the social domain can provide novel insights into the neural mechanisms underlying social interactions. In particular, a coordinate transformation approach to social interactions is useful for unraveling how neurons across different brain regions contribute to social interactions by framing their responses as cognitive states with respect to self and others.

## Coordinate transformation framework

A frame of reference refers to the coding scheme of a neuron representing information in specific coordinates (Groh, 2001; Cohen and Andersen, 2002). For example, a neuron is considered to use an eye-centered, or retinocentric, frame of reference when this neuron encodes a spatial location relative to a location on the retina (Batista et al., 1999; Avillac et al., 2005; Marzocchi et al., 2008; Chang and Snyder, 2010). This means that the receptive field of this neuron is anchored to the retinal location. On the contrary, a neuron may use an arm-centered reference frame when the neuron represents spatial location relative to a location on the arm (Kalaska et al., 1989; Caminiti et al., 1991; Scott and Kalaska, 1997; Schwartz et al., 2004; Batista et al., 2007; Chang and Snyder, 2010). Other documented reference frames include world-centered (information is encoded relative to a location in the world) (O'Keefe and Nadel, 1978; Snyder et al., 1998) and object-centered (relative to a certain feature of an object) (Olson and Gettner, 1995). It is important to note that not all reference frames are tightly coupled to specific body parts or well-defined location in the world, making some reference frames hard to interpret. For instance, some representations could be more accurately described as “intermediate,” that is, referenced to a position in between different body parts or different specific locations in the environment. Indeed, converging experimental evidence has documented such added complexity in neuronal reference frames (Mullette-Gillman, 2005; Chang and Snyder, 2010; McGuire and Sabes, 2011). Furthermore, depending on the goal of the transformation, there exists a final frame of reference for directly influencing a motor output. For instance, for visually-guided reaching, the representation eventually needs to be in an intrinsic muscle- or joint-centered frame of reference (Kalaska et al., 1989; Scott and Kalaska, 1997) in order to drive the arm at the end of the transformation pathway (Shadmehr and Wise, 2005).

**Figure 1:**
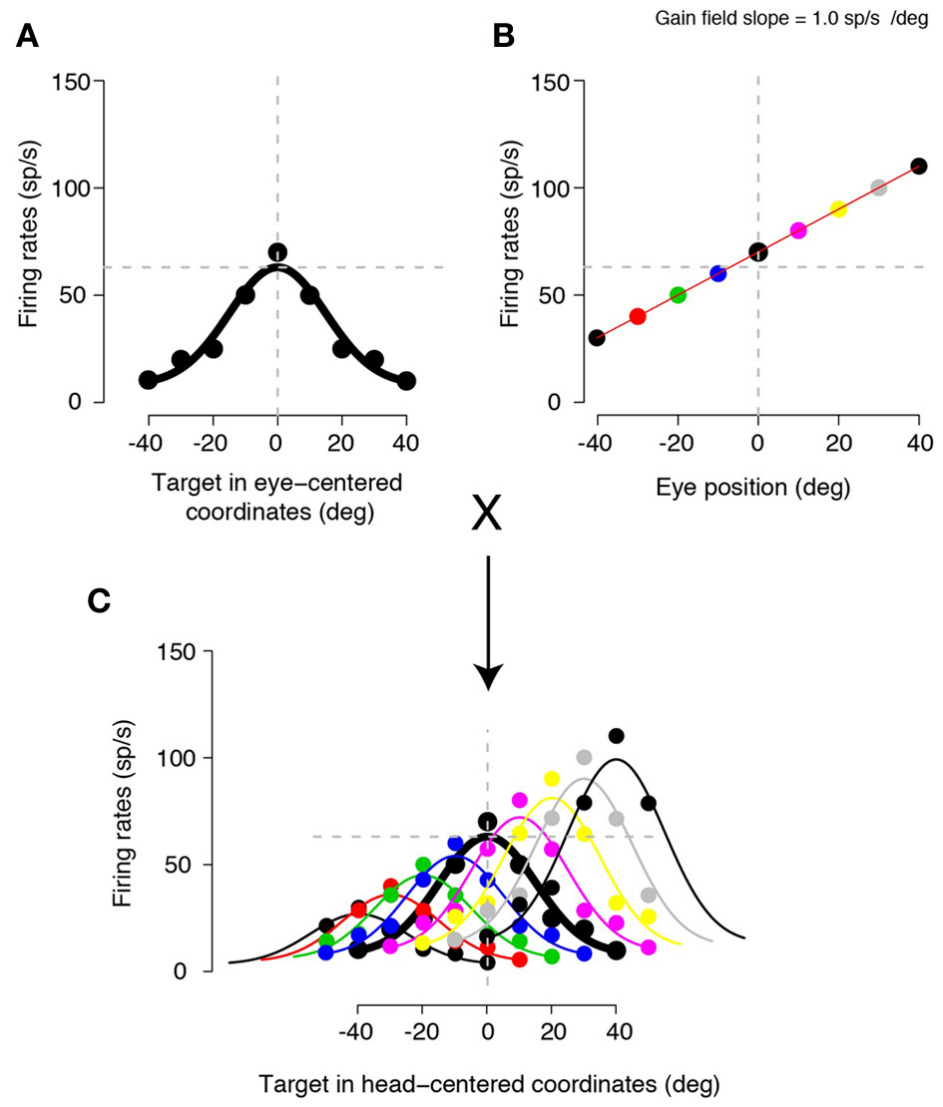
**A simulation of a gain field mediated coordinate transformation. (A)** A neuron encoding target locations using an eye-centered frame of reference, when the eyes are straight ahead. **(B)** The same neuron showing an eye position gain field. The responses are shown for a target straight ahead of and aligned with the eyes. The responses are monotonically scaled by changes in eye position (eyes-on-head). For this particular example neuron, the activity increases by 1.0 spikes/sec (sp/s) for each visual degree of rightward change in eye position. Different colored points represent different eye positions. **(C)** Multiplying the eye-centered tuning curve with different eye positions. Each colored curve represents a tuning curve obtained by multiplying the eye-centered tuning from **(A)** with each eye position from **(B)** (in corresponding colors). When the eye position is at 0, that is, the same position as the plot in **(A)**, the tuning does not change (indicated by the thick black traces in **A** and **C**). However, when the eye positions change, the tuning curves now scale and shift according to the eye position gain field. These multiplicative interactions result in target representations that can be read out in a head-centered frame of reference; now the responses are tuned relative to the head.

One of the powerful aspects of characterizing the reference frames employed by individual neurons is that it provides us with a relatively straightforward way to understand how different computational stages (roughly analogous to different brain areas) transform one type of a representation into another (Andersen et al., 1993; Pouget and Sejnowski, 1997; Pouget and Snyder, 2000; Snyder, 2000; Groh, 2001; Crawford, 2004). A next stage of computation might involve yet another coordinate transformation, depending on the purpose of the transformation (Andersen et al., 1993). A simulation in Figure 1 illustrates a popular example of coordinate transformation from an eye-centered to a head-centered frame of reference. This example computes the transformation using a gain field (i.e., multiplicative influence on neuronal tuning), which seems to be ubiquitously present across many brain regions (Salinas and Thier, 2000; Salinas and Sejnowski, 2001). Let us consider an eye-centered neuron (Figure 1A), like a neuron in area 7a (Andersen and Mountcastle, 1983), that monotonically modulates firing rates to changes in eye position (i.e., an eye position gain field, Figure 1B). When the eye-centered tuning is multiplied by the eye position gain field, a head-centered tuning begins to emerge (i.e., providing a basis for a population code that can be read out as head-centered) (Figure 1C). Various neural network models (Zipser and Andersen, 1988; Salinas and Abbott, 1996; Pouget and Snyder, 2000; Blohm et al., 2008) can efficiently perform this computation. If necessary for a given behavior, when a head-centered representation is multiplied by a head position gain field (Brotchie et al., 1995), yet another representation begins to emerge, namely a population code that can be read out as body-centered (Andersen et al., 1993; Snyder et al., 1998). Another example of coordinate transformation concerns directly converting (i.e., without the necessity of the serial steps as discussed in the previous example) an eye-centered representation of a reach target into an arm-centered representation by the reaching-related neurons. In the parietal reach region (PRR) of the primate posterior parietal cortex, this transformation can occur when the eye-centered representation of the hand, encoded using a compound eye and hand gain field specifying the distance between the eyes and the hand (Chang et al., 2009), is effectively vectorially subtracted from the eye-centered representation of the reach target, resulting in the hand-centered target representation (Bullock and Grossberg, 1988; Buneo et al., 2002; Chang et al., 2009).

## Selected theories of coordinate transformations

In this section, I will discuss two influential theories of coordinate transformation. By analogy, these contrasting theories can help guide how we interpret neuronal encoding and how such encoded variables are computed during social interactions. One theory focuses on systematic representations of neuronal variables (as in engineering a specific circuit based on a specific set of rules), whereas the other focuses on idiosyncratic neuronal representations (as in carrying out network-like operations using an artificial intelligence). For convenience, hereafter I will refer to them as the engineering approach and the connectionist approach, respectively.

From the classical engineering perspective, purpose-built networks are designed to compute highly specific quantities under strict rules. This engineering approach emphasizes that every neural representation serves a specific functional purpose using precise quantities. As a classic example, areas 7a neurons not only represent eye-centered target location but also show eye position gain fields (Andersen and Mountcastle, 1983), thereby providing a basis for a population code that can be read out as head-centered using a multiplicative interaction between eye-centered tuning and an eyes-on-head position signal (Figure 1) (Zipser and Andersen, 1988). Although such systematicity may restrict flexibility in creating novel representations for which the system is not initially designed to compute (but it remains unclear what the biological consequences might be), it is associated with extremely efficient computational performance.

On the contrary, an artificial intelligence field emphasizes the use of neural networks that contain multiple non-linear combinations of signals that are eventually self-organized in order to generate a particular information (Poggio, 1990). Such networks based on the connectionist approach have been successfully applied to perform coordinate transformations (Pouget and Sejnowski, 1997; Pouget and Snyder, 2000). Desired relationships of input and output variables may emerge from the hidden layer of such models (e.g., Chang et al., 2009). A connectionist approach suggests that diverse representations are common, and the vast majority of computations may appear highly obscure. Strong empirical evidence in support of the connectionist approach is the presence of intermediate neuronal representations. Intermediate reference frames, which are particular types of intermediary representations, are often desired for computational flexibility (Pouget and Sejnowski, 1997; Pouget and Snyder, 2000; Xing and Andersen, 2000; Blohm et al., 2008). Indeed, intermediate reference frames have been found across neurons in the lateral intraparietal area (LIP) (Mullette-Gillman, 2005), the ventral intraparietal area (VIP) (Avillac et al., 2005), PRR (Chang and Snyder, 2010), the dorsal area 5 (McGuire and Sabes, 2011), the dorsal medial superior temporal area (MSTd) (Fetsch et al., 2007), as well as the dorsal premotor cortex (PMd) (Batista et al., 2007). In exchange for high flexibility, such connectionist computations require high dimensional space, potentially demanding much more resources.

## Reference frames during social interactions

A successful social interaction requires an accurate understanding of self and others. Such representations of self and others can take many forms in the brain, including the agency underlying particular perceptual or emotional events (Ruby and Decety, 2004; Amodio and Frith, 2006; Mitchell et al., 2006; Singer, 2006; Ochsner et al., 2008), during action observation (Wolpert et al., 2003), and for learning and decision-making (Behrens et al., 2009). Here one can draw an analogy from the coordinate transformation framework, and apply it toward understanding the neural mechanisms of social interactions.

The analogy can be made based on the following criteria. First, as for representing sensory or motor information in a specific coordinate system for sensorimotor computations, representations of social information must be referenced to a specific agent (e.g., self, other, in-group, or out-group, etc.) involved in social interactions. Otherwise, normal social interactions simply would not be possible. So, the concept of reference frame is useful for social computations. Accumulating evidence suggests the presence of social reference frames during social behavior (Behrens et al., 2008; Yoshida et al., 2011, 2012; Chang et al., 2013). Second, similar to gain-modulated spatial representations during sensorimotor computations, social representations are systematically enhanced or attenuated according to behaviorally-relevant social variables (e.g., social status, familiarity). For example, studies have shown that social status and other social category modulate the gain of neuronal activity (Klein et al., 2008; Azzi et al., 2012; Watson and Platt, 2012). In this view, the concept of coordinate transformation using gain modulations could be analogously applied to social computations. Taken together, transforming spatial signals from one coordinate system to another is analogous to transforming agent-independent signals into agent-specific signals, or converting signals referenced to one type of agent to another.

In what way can neuronal variables represented during social interactions be considered as having reference frames? Let us consider a simple scenario in which two individuals, agent A and agent B, are playing an afternoon chess at a park. For every move that is made, agent A needs to keep track of the actions of both himself and agent B as well as the outcomes for themselves resulting from each move. Agent B also does the same to have a chance at winning. These actions and outcomes tightly coupled to either agent A or B during their competitive exchanges must be reflected in their neuronal signals. More precisely, these variables with respect to self and others need to be either differentiated or coincided during different stages of computations. Although the above example focused on a competitive interaction, tracking self and others' actions and outcomes is similarly importantly for cooperative transactions, such as when agents A and B need to coordinate steering to the right on a canoe to avoid a rock in their way. Furthermore, it is natural to consider that inaccurate or unstable representations of social variables across self- and other-centered frames of reference may directly underlie many of the social deficits observed in multiple psychiatric conditions (see below). It is worthwhile to emphasize, however, that applying the coordinate transformation framework based on spatial reference frames to cognitive domains is an analogy by nature simply because cognitive computations, like those involved in social cognition, are fundamentally different from the sensorimotor computations using the receptive field or place code. Rather, the analogy is beneficial for understanding how social variables represented in different *dimensions* (e.g., self versus others) are used to mediate social interactions.

Reward-guided social learning and decision-making have been critical for investigating neural basis of social behaviors (King-Casas et al., 2005; Moll et al., 2006; Behrens et al., 2008, 2009; Mobbs et al., 2009; Jeon et al., 2010; Yoshida et al., 2011, 2012; Azzi et al., 2012; Carter et al., 2012; Hillman and Bilkey, 2012; Kishida and Montague, 2012; Nicolle et al., 2012; Watson and Platt, 2012; Chang et al., 2013). Given that social interactions are largely reward-driven (Fehr and Camerer, 2007), it is not surprising that self- and other-referenced signals are robustly present in reward-related brain regions. Taking inspiration from work in reinforcement learning (Sutton and Barto, 1998), vicarious reinforcement (Berber, 1962; Bandura et al., 1963), neuroeconomics (Platt and Huettel, 2008), and game theory (Lee, 2008), researchers have begun the quest to identify neural correlates of social learning and decision-making (Sanfey, 2007; Behrens et al., 2009; Seo and Lee, 2012; Rushworth et al., 2013). One common goal for this expedition has been to elucidate how different brain regions compute social variables with respect to self and others. Another shared aim of this quest, which will not be discussed here, has been to identify whether there are neural circuits dedicated to social cognition (Carter et al., 2012; Rushworth et al., 2013).

**Figure 2:**
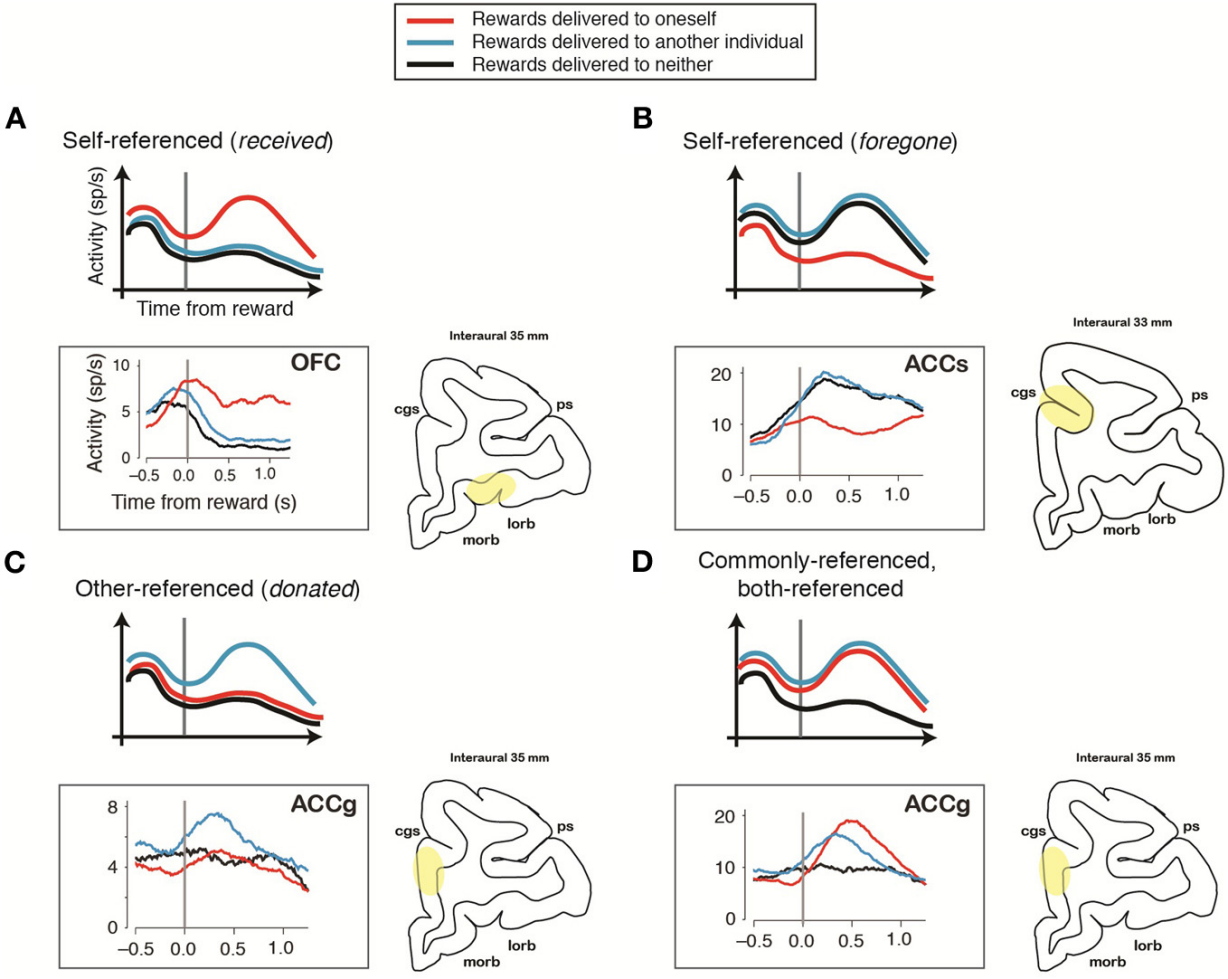
**Schematic and empirical examples of reward outcomes represented in different frames of reference during social interactions.** Illustrative peri-stimulus time histograms (PSTHs) (top of each panel) show the activity of an individual reward-sensitive neuron aligned to the time of reward. The PSTHs displayed on the bottom of each panel (in the gray box) show the activity of a single neuron recorded from different regions of the primate frontal cortex during a social reward-allocation task [modified with permission from Chang et al. (2013)] that corresponds to the illustrative PSTHs above. The brain region from which each neuron was recorded is highlighted on the right (in yellow). cgs, cingulate sulcus; lorb, lateral orbitofrontal sulcus; morb, medial orbitofrontal sulcus; ps, principal sulcus. **(A)** Self-referenced representation of actor's received rewards. The majority of the orbitofrontal cortex (OFC) neurons employ this coding scheme. **(B)** Self-referenced representation of actor's foregone rewards. The majority of neurons located in the sulcus of the anterior cingulate cortex (ACCs) employ this coding scheme. **(C)** Other-referenced representation of rewards allocated to another monkey in the room. A group of neurons in the gyrus of the anterior cingulate cortex (ACCg) employs this coding scheme. **(D)** Common (both-referenced) representation of rewards received by an actor and another monkey. A group of ACCg neurons employs this coding scheme.

Recent studies are beginning to unravel how self- and other-referenced computations are computed across multiple brain regions. Using behavioral tasks involving interacting rhesus monkeys, single-neuron recording studies from reward-sensitive areas, such as the anterior cingulate gyrus (ACCg), anterior cingulate sulcus (ACCs), orbitofrontal (OFC) cortices, and the regions in the medial frontal cortex (MFC), have characterized how individual neurons modulate activity with respect to events occurring to self and others (Yoshida et al., 2011, 2012; Azzi et al., 2012; Chang et al., 2013). Yoshida and colleagues reported that a group of primate MFC neurons selectively encode actions in other-centered frame of reference (Yoshida et al., 2011), and that some MFC neurons encode self-referenced reward-omission signals or other-referenced error signals (others' erroneous actions) (Yoshida et al., 2012). Azzi and colleagues reported that primate OFC neurons modulate activity according to whether rewards are shared with another monkey or received only by the actor monkey (Azzi et al., 2012). Using fully dissociated self and other reward outcomes, Chang and colleagues reported that primate OFC neurons signal actors' received rewards in a self-centered frame of reference (Figure 2A), whereas ACCs neurons signal actors' foregone rewards (rewards that are either omitted or delivered to another) in a self-centered frame of reference (Figure 2B) (Chang et al., 2013). In contrast, in addition to OFC-like self-referenced reward neurons, some ACCg neurons selectively signal others' received rewards in other-centered frame of reference (Figure 2C), while others signal actors' received and others' received rewards in a common, or both-centered, frame of reference (Figure 2D) (Chang et al., 2013). Furthermore, in humans, Nicolle and colleagues reported that self- and other-referenced decision signals in the ventromedial prefrontal cortex (vmPFC) and dorsomedial prefrontal cortex (dmPFC) flexibly switch their coding schemes such that vmPFC always track relevant choices (for whom a choice is being made) and dmPFC always track irrelevant choices (for whom a choice is not being made) (Nicolle et al., 2012). Together, these results provide novel intuitions into how different neural circuits encode self- and other-referenced information during social interactions. At the same time, they highlight that the remarkable flexibility in transformations across the two representations, depending on task demands.

## Applying the coordinate transformation framework to social interactions

**Figure 3:**
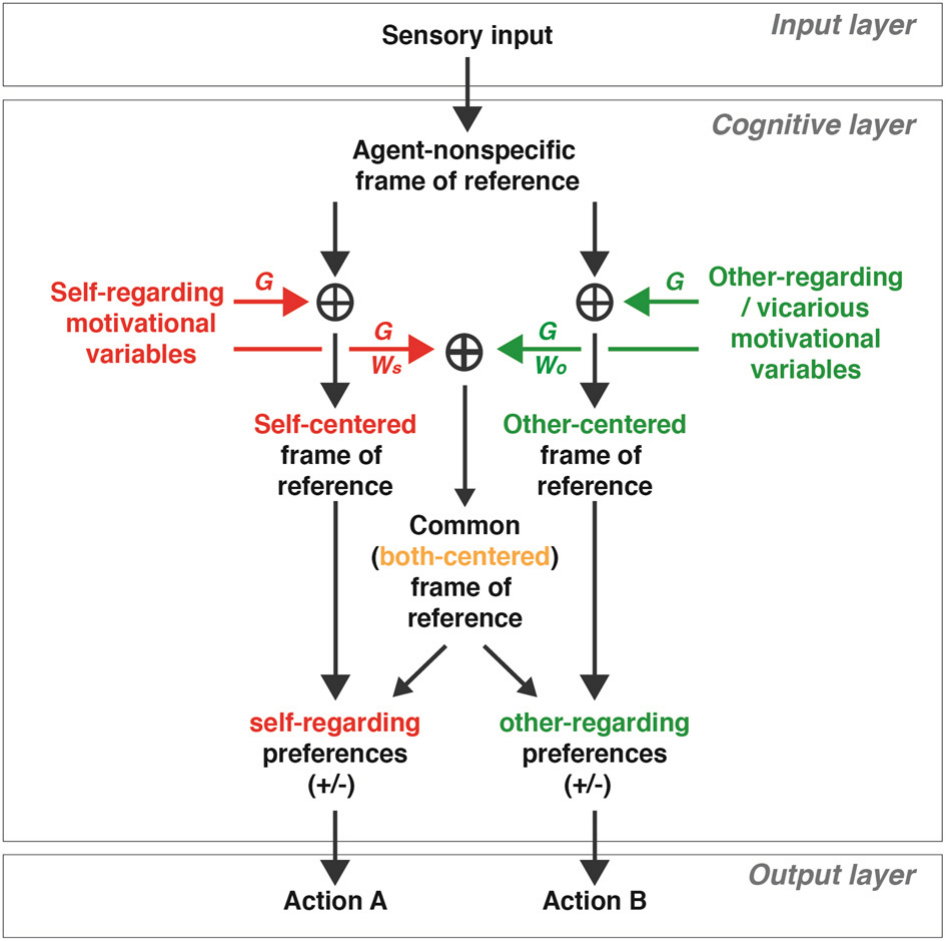
**A proposed schematic model of how social variables represented in self- and other-centered, as well as common (both-centered), frames of reference may mediate social interactions.** In the cognitive layer, neuronal signals resulting from the environment (input layer) are represented in an agent-nonspecific frame of reference. Motivational (and other cognitive) signals regarding oneself (self motivation variables; see examples in the text) can be added using gain modulations (*G*) to generate a representation in a self-centered frame of reference, whereas motivational (and other cognitive) signals regarding others (other-regarding and vicarious motivation variables; see examples in the text) can be added using gain modulations to generate a representation in an other-centered frame of reference. Neuromodulators (see examples in the text) sets the gain parameters (e.g., magnitude, context) of self- and other-regarding motivational signals in a context-dependent manner. Both self- and other-regarding motivational signals can be added together using gain modulations in a weighted manner (*W*_*S*_ and *W*_*O*_, respectively) to result in a representation in a common (both-centered) frame of reference. The relative distribution of *W*_*S*_ and *W*_*O*_ determines the strength of self- and other-regarding signals for the both-centered representation. The self-centered signals directly influence self-regarding preferences (either positive or negative in valence, +/−), whereas the other-centered signals directly influence other-regarding preferences (either positive or negative in valence). On the other hand, the commonly-referenced, both-centered, signals may influence the self- and other-regarding preferences, and the strength of each influence depends on *W*_*S*_ and *W*_*O*_. The self- and other-regarding preference signals are relayed to the output layer to generate different social decisions and actions.

A proposed schematic model in Figure 3 illustrates how self-referenced, other-referenced, and commonly-referenced (both-referenced) signals may arise from coordinate transformations during social interactions. This model, like the models used for the coordinate transformations for sensorimotor behaviors (Zipser and Andersen, 1988; Salinas and Abbott, 1996; Blohm et al., 2008; Chang et al., 2009), utilizes gain modulations (noted as *G* in Figure 3) to transform signals represented in an agent-nonspecific coordinate to a coordinate with respect to self, other, or both. For example, added gain modulations based on a variety of self motivational variables can result in a self-referenced representation, as reported in the primate OFC (actors' received rewards), ACCs (actors' foregone rewards), and a subgroup of ACCg neurons (actors' received rewards) (Chang et al., 2013). On the other hand, added gain modulations based on other-regarding variables can result in selectively other-referenced reward signals, like those documented in a subgroup of ACCg neurons (Chang et al., 2013), and other-referenced action and error signals, as reported in MFC neurons (Yoshida et al., 2011, 2012). Examples of self-regarding motivational variables include reward amount, risk, uncertainty, expected utility, delay, and so on. In contrast, examples of other-regarding motivational variables include social relationship, reciprocity level, trustworthiness, generosity, and so forth, in addition to the variables like those that drive self-motivation but directed toward others. It is important to note that social variables such as social relationship, reciprocity level, trustworthiness, and generosity may also contain self-regarding components since self motivations sometimes underlie other-regarding motivations (e.g., Weinstein and Ryan, 2010). Thus, the signals that drive other-regarding gain in the model should correspond to *other-referenced components* of such complex social variables.

Furthermore, for generating a both-referenced representation, the model assigns appropriate weights for self motivations (noted as *W*_*S*_) and for other-regarding motivations (*W*_*O*_) to account for the different strengths of modulations with respect to self and others. This relative weighting offers a modulatory control over both-centered representations. For instance, when the two weights are equal (*W*_*S*_ = *W*_*O*_), the signals with respect to self and other in the both-centered representations will appear to be mirrored. In contrast, a greater influence of self motivational signals (*W*_*S*_ > *W*_*O*_) will result in a stronger representation for the signals with respect to self in the both-centered representation, whereas the opposite pattern is apparent when there is a greater influence of other-regarding motivational signals (*W*_*S*_ < *W*_*O*_). Such computations may result in differentially modulated activity corresponding to different social contexts, perhaps similar to what has been reported in OFC neurons (Azzi et al., 2012).

Neuromodulators, such as oxytocin, norepinephrine, dopamine, and testosterone, may set the gain parameters (e.g., magnitude, context) (Servan-Schreiber et al., 1990; Fellous and Linster, 1998) of the self- and other-regarding variables in a context-dependent manner. Neuromodulators therefore may directly gate when and how much of gain modulations are taking place across different neural circuits (Dayan, 2012) for both social and nonsocial behaviors. For instance, oxytocin, known for its role in modulating social cognition (Donaldson and Young, 2008), amplifies both self and vicarious reinforcement (increases both red and green Gs in Figure 3) in rhesus monkeys during social decision-making in a context-dependent manner (Chang et al., 2012). It is worthwhile to emphasize that neuromodulator action could be one of many ways to adjust the gain parameters during social interactions. Furthermore, it is expected that Gs in the model are sensitive to social context signals, and different Gs might be independently controlled by multiple sources. In this regard, the temporal dynamics of neuromodulator-dependent gain control is important to consider. In typical social interactions, it is often necessary for neuronal representations of social variables (e.g., who is being rewarded for a particular action) to alternate rapidly between being referenced to self and another individual. Such fast dynamics for rapid and flexible updating are likely to be mediated by gain modulations by fast neurotransmission (e.g., via AMPA or GABA receptors) or slightly slower (order of seconds) G-protein-coupled neuromodulator action (e.g., oxytocin or vasopressin). In contrast, an overall social state of an individual (e.g., prosocial or antisocial tendency), whether it is typical or pathological (e.g., attenuated social motivation in autism; see Chevallier et al., 2012), is likely to change much more slowly by comparison. Such longer-term dynamics are likely to be mediated by an overall up- or down-regulation of neuromodulators and their receptors. Finally, it is critical to point out that certain neuromodulators, like dopamine, are involved in both fast and slow time scale depending on its functional contribution to behavior (Schultz, 2007).

Similar to the heterogeneity of reference frames found for sensorimotor behaviors (Mullette-Gillman, 2005; Chang and Snyder, 2010; McGuire and Sabes, 2011), it is likely that some brain regions may concurrently represent social variables using multiple frames of reference. For instance, neural networks within a given area may activate multiple pathways in the model. The mixed self-, other-, and both-referenced social reward signals found in ACCg support this view (Chang et al., 2013). However, other areas like ACCs, which encodes actors' foregone rewards in a self-centered reference frame (Chang et al., 2013), seem to represent information in a unified single frame of reference. This might be analogous to some sensorimotor regions representing information primarily using a single frame of reference (e.g., eye-centered tuning with an eye position gain field in the primate V4; Bremmer, 2000). Furthermore, coding of information in intermediate social reference frames is likely to be present for computational flexibility. Finally, as in sensorimotor transformations, social coordinate transformations might occur in multiple directions. For example, self-referenced variables could be transformed into other- or both-referenced variables, and vice versa. Such flexibility, perhaps mediated by intermediate social reference frames and gain modulations, would be beneficial for rapidly updating representations across different social reference frames.

## Insights for social computation from coordinate transformation theories

As mentioned in the earlier section, the engineering and the connectionist approaches describe how neuronal variables are encoded and how they are being computed to result in a desired output during sensorimotor behavior. These two theoretical frameworks could be useful for characterizing how social variables are encoded across different brain regions or different computational stages. For example, highly systematic representations of social variables would suggest that the region serves a specific functional purpose using well-defined social quantities to maximize efficiency. For instance, neurons in the population might be tuned to social status using a shared encoding principle. Under this encoding, population average is particularly meaningful (e.g., preferred direction encoding by individual neurons and population vector averaging for movement direction representations; e.g., Georgopoulos et al., 1986). Alternatively, highly idiosyncratic representations of social variables by a heterogeneous population would instead suggest that the social computations in this region rely on complex non-linear combinations of signals taking place in a high dimensional space to maximize flexibility. For example, individual neurons in a population might encode diverse, seemingly random permutations of social status information, rendering a standard population pooling problematic. As in the computations of sensorimotor behavior across different brain areas, it is likely that distinct neural circuits employ different computational strategies for mediating social interactions.

## Coordinating self- and other-referenced representations: implications for social deficits in psychopathology

A strikingly large number of neuropsychiatric disorders are accompanied by social deficits (Insel, 2010; Meyer-Lindenberg and Tost, 2012). Many of which are believed to be rooted in an inability to appropriately understand representations of self and others. Atypical social behaviors in autism (Rogers and Pennington, 1991; Charman, 2003; Dawson et al., 2004; Lombardo et al., 2009), schizophrenia (Jeannerod, 2008), borderline personality disorders (Bender and Skodol, 2007), psychopathy (Hare, 1999), among others, seem to have an underlying impairment in coordinating self and other representations. For example, deficits in self-referential and other-referential processing in individuals with autism are reflected in an inability of the ventromedial prefrontal cortex (vmPFC) to robustly differentiate mentalizing about self and others (Lombardo et al., 2009). Furthermore, in schizophrenia, many psychotic episodes are thought to originate from a deficit in monitoring other-referenced action (other's behavior) and relating one's own intention to self-referenced action (one's own behavior) (Brune, 2005). Misalignments in these representations and inabilities to dynamically switch across different reference frames can ultimately result in deficits in empathy and theory of mind (Brüne and Brüne-Cohrs, 2006). Depending on the precise type of psychopathology, such misalignments may be originating from sensory (Lindner et al., 2005), motor (McIntosh et al., 2006), or motivational and other cognitive modalities (Chevallier et al., 2012).

The model in Figure 3 generates several testable hypotheses for social deficits in psychopathological states. Unbalanced self- and other-regarding preferences may result from overactive or underactive gain modulations used for transforming agent-nonspecific signals to either self- or other-referenced signals (*G* **in** Figure 3). They could also result from, or further worsened by, an inability to appropriately assign the relative contributions (*W*_*S*_ and *W*_*O*_ in Figure 3) of self- and other-regarding motivational variables for generating a both-referenced representation. Such differential weighting might be particularly relevant during cooperative interactions in which commonly referenced computations might be crucial. Empirically testing these and other hypotheses over time will help validate, refine, or reject the details of the model.

## Concluding remarks

A successful social interaction requires one to track the behaviors of oneself as well as the behaviors of another individual, requiring the brain to integrate both motivational and affective variables across interacting individuals (Schilbach et al., 2013). In this article, I proposed a coordinate transformation approach toward understanding the neural mechanisms of social interactions. This approach, borrowed from the sensorimotor tradition, can provide a computational framework for investigating the representations of self and others in both healthy and psychopathological brains. A particular advantage of this approach over others is that the social coordinate transformation model focuses on how social variables are *encoded* by individual neurons, and how such encoding may *evolve* across different computational stages. Therefore, the coordinate transformation approach for social interactions may provide valuable insights into how social information used within various computational models, such as reinforcement learning and game theoretic models, is encoded and transformed across different processing stages. Applied in conjunction with the reinforcement learning framework, it may be especially useful for revealing how the brain assigns reward outcomes across different agencies during social interactions. A bright future lies ahead for social neuroscience. We are now well poised to the test different social psychological theories by directly investigating neural mechanisms. An influential computational scaffold like the coordinate transformation framework will help advance our understanding of social cognition, for which the brains of humans and nonhuman primates have evolved to be specialized.

### Conflict of interest statement

The author declares that the research was conducted in the absence of any commercial or financial relationships that could be construed as a potential conflict of interest.
